# Physical Localization of a Locus from *Agropyron cristatum* Conferring Resistance to Stripe Rust in Common Wheat

**DOI:** 10.3390/ijms18112403

**Published:** 2017-11-13

**Authors:** Zhi Zhang, Liqiang Song, Haiming Han, Shenghui Zhou, Jinpeng Zhang, Xinming Yang, Xiuquan Li, Weihua Liu, Lihui Li

**Affiliations:** 1 National Key Facility for Crop Gene Resources and Genetic Improvement, Institute of Crop Sciences, Chinese Academy of Agricultural Sciences, Beijing 100081, China; zhangzhihkd@126.com (Z.Z.); hanhaiming@caas.cn (H.H.); zhoushenghui@caas.cn (S.Z.); zhangjinpeng@caas.cn (J.Z.); yangxinming@caas.cn (X.Y.); lixiuquan@caas.cn (X.L.); liuweihua@caas.cn (W.L.); 2 Center for Agricultural Resources Research, Institute of Genetics and Developmental Biology of Sciences, Shijiazhuang 050022, China; songliqiang.1988@163.com

**Keywords:** stripe rust, *A*. *cristatum*, common wheat, translocation lines

## Abstract

Stripe rust, caused by *Puccinia striiformis* f. sp. *tritici* (*Pst*), is one of the most destructive diseases of wheat (*Triticum aestivum* L.) worldwide. *Agropyron cristatum* (L.) Gaertn. (2*n* = 28, PPPP), one of the wild relatives of wheat, exhibits resistance to stripe rust. In this study, wheat-*A*. *cristatum* 6P disomic addition line 4844-12 also exhibited resistance to stripe rust. To identify the stripe rust resistance locus from *A*. *cristatum* 6P, ten translocation lines, five deletion lines and the BC_2_F_2_ and BC_3_F_2_ populations of two wheat-*A*. *cristatum* 6P whole-arm translocation lines were tested with a mixture of two races of *Pst* in two sites during 2015–2016 and 2016–2017, being genotyped with genomic in situ hybridization (GISH) and molecular markers. The result indicated that the locus conferring stripe rust resistance was located on the terminal 20% of 6P short arm’s length. Twenty-nine 6P-specific sequence-tagged-site (STS) markers mapped on the resistance locus have been acquired, which will be helpful for the fine mapping of the stripe rust resistance locus. The stripe rust-resistant translocation lines were found to carry some favorable agronomic traits, which could facilitate their use in wheat improvement. Collectively, the stripe rust resistance locus from *A*. *cristatum* 6P could be a novel resistance source and the screened stripe rust-resistant materials will be valuable for wheat disease breeding.

## 1. Introduction

Stripe rust, caused by *Puccinia striiformis* f. sp. *tritici* (*Pst*), is one of the most devastating and widespread diseases of wheat (*Triticum aestivum* L.) around the world [1,2,3]. Stripe rust has become a major threat to wheat production, causing yield losses of 5–25% [4,5]. In recent years, new virulent *Pst* races appeared in a short period of time so that many wheat varieties were ineffective against prevalent races [6]. Therefore, there is a need to screen new stripe rust resistance genes for broadening the wheat gene pool and providing new potential resistance genes for the wheat breeding of stripe rust resistance.

Wide hybridization is an efficient way of transferring beneficial resistance genes to common wheat. Different resistance genes from distant genera, such as *Yr9* from *Secale cereal* [7,8], *Pm21* from *Haynaldia villosa* [9] and *YrH9020* from *Psathyrostachys huashanica* [10], have been transferred to common wheat, and were effective in enhancing the disease resistance of wheat. The disease-resistant wheat-alien species derivative lines will be used as new wheat resources for breeding new resistant varieties.

The genus *Agropyron* (Gaertn.) belongs to the tribe Triticeae and is based on the P genome. *Agropyron cristatum* (L.) Gaertner, the most common species, has diploid (2*n* = 2*x* = 14, PP), tetraploid (2*n* = 4*x* = 28, PPPP) and hexaploid (2*n* = 6*x* = 42, PPPPPP) forms. Tetraploid *A*. *cristatum* carries yield-related genes [11,12,13] and biotic and abiotic resistance genes [14,15,16], which is a favorable genetic resource for the genetic improvement of wheat. With the accomplishment of the wide hybridization of common wheat and *A*. *cristatum* [17,18,19], many desirable genes from *A*. *cristatum* have been transferred into common wheat. Wu et al. [20] found that *A*. *cristatum* 6P carried multi-kernel gene(s) [20]. Lu et al. [21] localized the higher thousand-grain weight locus on 7PS arm using translocation lines and deletion lines. Li et al. [22] and Copete et al. [23] confirmed that 2PL arm carried powdery mildew resistance gene(s), meanwhile Copete et al. [23] also found that 6PL arm carried powdery mildew resistance gene(s) using the addition lines. Ochoa et al. [16] have acquired the leaf rust-resistant wheat-*A*. *cristatum* translocation line.

To map alien desirable genes, a series of wheat-alien translocation lines and deletion lines were produced to construct the physical map. Qi et al. [24] constructed the physical mapping of *Haynaldia villosa* 6V using one spontaneous deletion line and two deletion lines induced by irradiation. Ashida et al. [25] created chromosomal breaks in the offspring of a 5H addition line induced by the gametocidal chromosome and constructed the physical map of barley chromosome 5H. Wheat-*A*. *cristatum* translocation lines and deletion lines have been developed through the irradiation of wheat-*A*. *cristatum* disomic addition lines, and high-resolution physical maps of *A*. *cristatum* 2P, 6P and 7P have been constructed [21,22,26,27,28,29]. The physical maps and the introgression lines can provide a solid foundation for the exploration and utilization of elite genes from the wide relatives of wheat.

In this study, wheat-*A*. *cristatum* 6P derivative lines and the populations were used to map the stripe rust resistance locus from *A*. *cristatum* 6P to the specific chromosomal region. The resistance locus could be a novel stripe rust-resistant source and the screened resistant materials will be valuable for wheat disease breeding.

## 2. Result

### 2.1. Evaluation of Stripe Rust Resistance of Wheat-A. cristatum 6P Disomic Addition Line 4844-12

**Figure 1:**
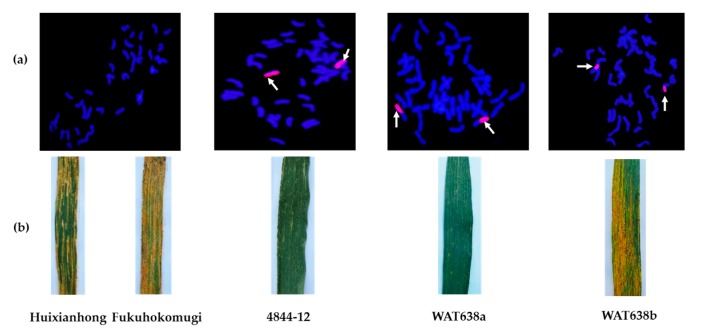
(**a**) GISH detection for the control Fukuhokomugi and the lines 4844-12, WAT638a and WAT638b containing intact 6P, 6PS and 6PL, respectively. *A*. *cristatum* chromosomal segments were in red, while wheat chromosomes were in blue strained by DAPI. (**b**) Evaluation of stripe rust of Huixianhong, Fukuhokomugi, 4844-12, WAT638a and WAT638b.

During 2015–2016 and 2016–2017, a mixture of prevalent physiological races (CYR32 and CYR33) was utilized to infect wheat-*A*. *cristatum* 6P disomic addition line 4844-12, common wheat “Fukuhokomugi” and common wheat “Huixianhong” in Yangling (Shaanxi province, China) and Xinxiang (Henan province, China). Among these materials, the line 4844-12 was highly resistant to stripe rust, while the controls Fukuhokomugi and Huixianhong were highly susceptible (Figure 1). Therefore, the locus conferring stripe rust resistance was derived from *A*. *cristatum* chromosome 6P.

### 2.2. Molecular Cytogenetic Analysis and Chromosomal Arm Localization of the Locus Conferring Resistance to Stripe Rust

**Figure 2:**
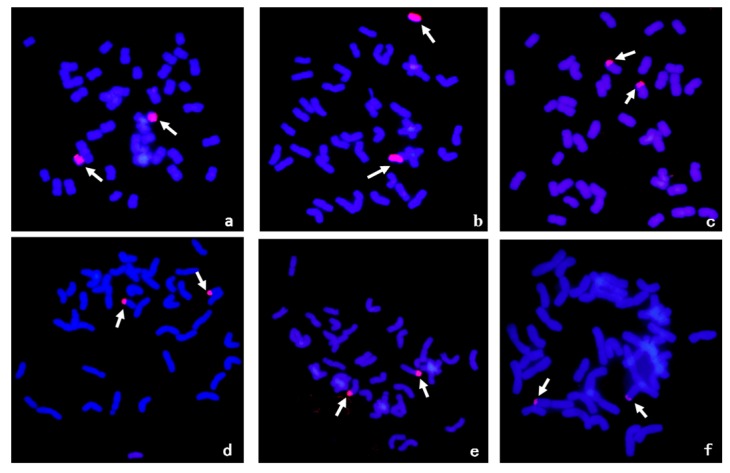
GISH patterns of wheat-*A*. *cristatum* 6P homozygous strains. *A*. *cristatum* chromosomes were in red, while wheat chromosomes were in blue strained by DAPI. (**a**) del21; (**b**) del19a; (**c**) WAT641a; (**d**) WAT657; (**e**) WAT648; and (**f**) WAT646. The homozygous materials carried a pair of chromosomal segments.

**Figure 3:**
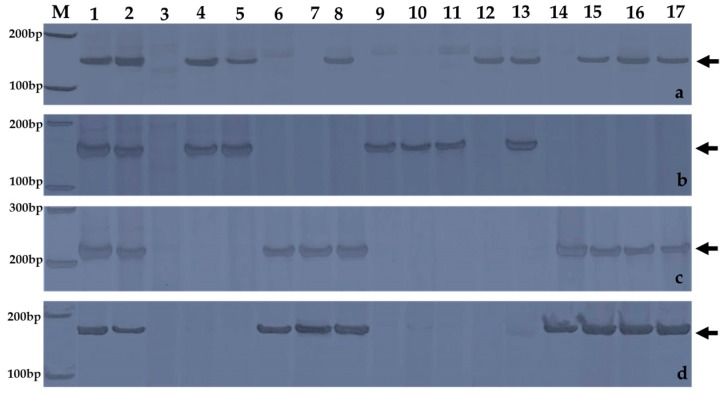
Amplification patterns of wheat-*A*. *cristatum* homozygous materials using 6P-specific STS markers: (**a**) Agc4527; (**b**) Agc37790; (**c**) Agc21670; (**d**) Agc2970. Lanes: M, DNA marker I; 1, *A*. *cristatum* accession Z559; 2, 4844-12; 3, Fukuhokomugi; 4, WAT639b; 5, WAT638a; 6, WAT639a; 7, WAT638b; 8, WAT644; 9, WAT655; 10, WAT648; 11, WAT641a; 12, WAT657; 13, del21; 14, del20; 15, del29; 16, del23; 17, del19a. Arrows indicated the diagnostic bands.

A series of wheat-*A*. *cristatum* 6P translocation lines and deletion lines have been developed through irradiation of the addition line 4844-12 [26,29]. Ten translocation lines and five deletion lines were used to map the stripe rust resistance locus. All of these lines contained a pair of segments of *A*. *cristatum* 6P detected by GISH (Figure 2). *A*. *cristatum* 6P-specific STS molecular markers were used to trace different chromosomal segments of *A*. *cristatum* 6P to confirm the constitutions of P chromatin in each line (Figure 3).

To identify the arm carrying the resistance locus, four wheat-*A*. *cristatum* 6P whole-arm translocation lines (WAT639b, WAT638a, WAT639a and WAT638b) and two 6P ditelosomic addition lines (del21 and del20) were tested using a mixture of the *Pst* races in two sites. Among them, all lines carrying chromosome arm 6PS were resistant; all lines carrying chromosome arm 6PL were susceptible (Table 1, Figure 1). This identifies the short arm of 6P as the carrier of the resistance locus.

**Figure 4:**
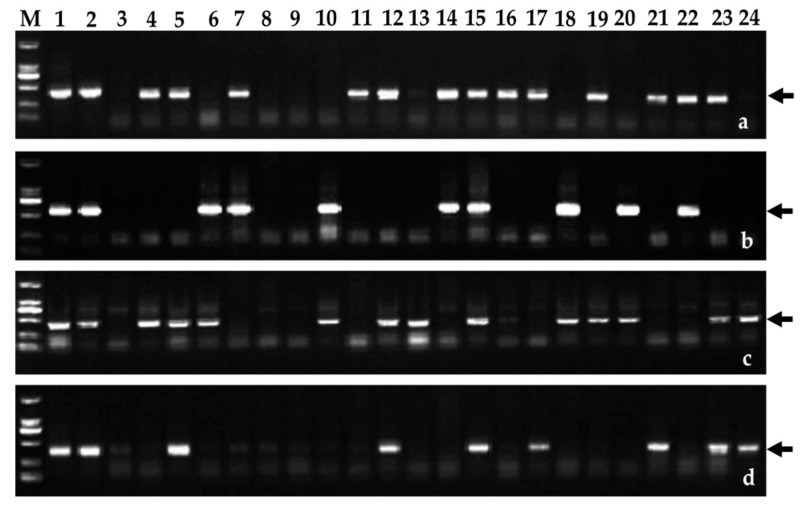
Amplification patterns of the BC_3_F_2_ population of wheat-*A*. *cristatum* 6P short-arm translocation lines WAT639b using P genome-specific molecular markers, including (**a**) AcPR6; (**b**) AcPR7; (**c**) AcPR3a; and (**d**) AcPR2a. Lanes: M, DL2000 DNA Marker; 1, Z559; 2, 4844-12; 3, Fukuhokomugi; 4–24, the partial plants of the WAT639b population. Arrows indicated the diagnostic bands.

The BC_2_F_2_ and BC_3_F_2_ populations of WAT639a and WAT639b were constructed to confirm the resistance locus on chromosome 6PS. P genome-specific molecular markers [30] were used to detect the populations (Figure 4). In the BC_2_F_2_ population (2015–2016 season) of the line WAT639b, 22 plants carried 6PS arm, which were resistant; while 32 did not carried 6PS, which were susceptible. In the BC_3_F_2_ population (2016–2017 season) of the line WAT639b, 97 plants carried 6PS arm, which were resistant; while 52 did not carry 6PS, which were susceptible (Table 2). Chi squared (*χ*^2^) tests for independence showed that stripe rust resistance was significantly affected by the 6PS arm. In the populations of the line WAT639a, no matter whether the plants contained the 6PL arm or not, all were susceptible, suggesting that there was no correlation between stripe rust resistance and the 6PL arm. Therefore, the 6PS arm surely carried the resistance locus.

### 2.3. Chromosomal Segmental Localization of the Stripe Rust Resistance Locus

**Figure 5:**
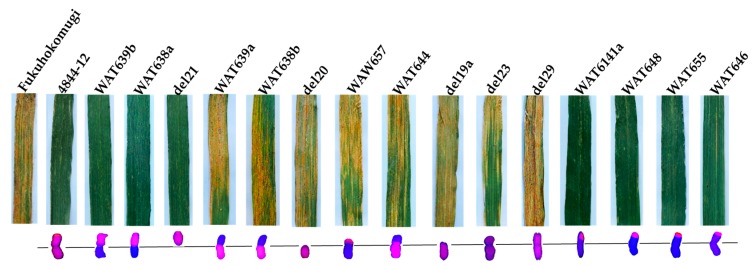
Evaluation of stripe rust resistance of wheat-*A*. *cristatum* 6P derivatives. GISH patterns: 6P chromosomal segments were in red, while wheat chromosomal segments were in blue strained by DAPI.

**Figure 6:**
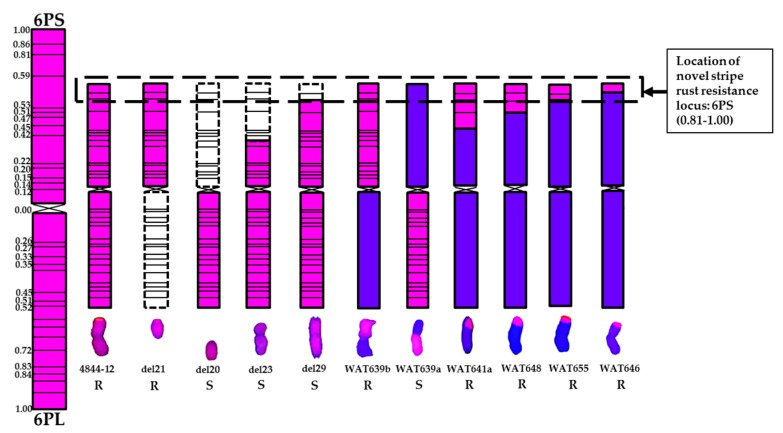
Chromosomal localization of the novel stripe rust resistance locus from *A*. *cristatum* 6P and chromosome diagrams of the deleted and the translocated 6P chromosomes. The left map showed that the diagram of *A. cristatum* chromosome 6P, which was modified in the physical map of *A*. *cristatum* 6P as described by Song et al. [29]. Pink and blue colors represented *A*. *cristatum* and wheat chromosomal segments, respectively. Dotted line boxes indicated the missing segments of 6P chromosome. The letters R and S indicated materials were resistant and susceptible, respectively.

Six translocation lines (WAT657, WAT644, WAT641a, WAT648, WAT655 and WAT646) and three terminal deletion lines (del19a, del23 and del29) were applied to further map the resistance locus to the smaller segment of 6PS arm. The lines WAT657, WAT644, del19a, del23 and del29 were highly susceptible to stripe rust (Figure 5, Table 1). They contained the segments 6PS (0.00–0.15), 6PS (0.00–0.20) + 6PL, 6PS (0.00–0.15) + 6PL, 6PS (0.00–0.45) + 6PL and 6PS (0.00–0.81) + 6PL, respectively, suggesting that the stripe rust resistance locus was not on 6PS (0.00–0.81). The terminal translocation lines WAT641a, WAT648, WAT655 and WAT646 carried the segments 6PS (0.53–1.00), 6PS (0.59–1.00), 6PS (0.81–1.00) and 6PS (0.86–1.00), respectively, which were highly resistant to stripe rust (Figure 5). Among the resistant lines, the leaves of WAT646 carried few spores of *Pst*. Therefore, we localized the resistance locus on the bin 6PS (0.81–1.00) of WAT655 (Figure 6).

### 2.4. Evaluation of Agronomic Traits of the Stripe Rust-Resistant Translocation Lines

**Figure 7:**
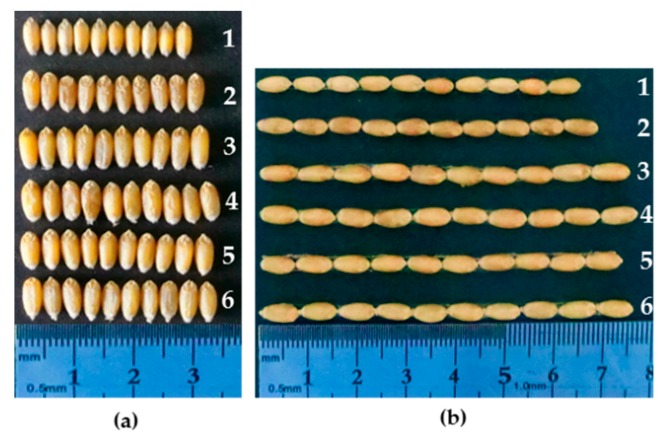
Morphologies of (**a**) grain width and (**b**) grain length. 1, Fukuhokomugi; 2, 4844-12; 3, WAT638a; 4, WAT648; 5, WAT655; and 6, WAT646.

The spike agronomic traits of six wheat-*A*. *cristatum* 6P translocation lines (WAT638a, WAT639b, WAT641a, WAT648, WAT655 and WAT646) conferring stripe rust resistance were evaluated at BC_2_F_3_ progeny, including spike length, spikelet number per spike, kernel number per spikelet, grain number per spike and thousand-grain weight in this study (Table 3). The seeds of the lines WAT638a, WAT639b, WAT648, WAT655 and WAT646 displayed longer and wider size than that of Fukuhokomugi (Figure 7), so that these translocation lines exhibited higher thousand-grain weights. Compared to Fukuhokomugi, the lines WAT655 and WAT646 showed higher grain number per spike, contributed by the higher spikelet number per spike based on Duncan’s multiple-range test.

## 3. Discussion

Broadening the genetic base of common wheat by transferring resistance genes from wide relatives may enhance the chance of achieving adequate resistance against stripe rust. Wheat-*A*. *cristatum* 6P disomic addition line 4844-12 was immune to the infection with stripe rust (CYR32 and CYR33). Six translocation lines WAT638a, WAT639b, WAT641a, WAT648, WAT655 and WAT646 were highly resistant to stripe rust. Among these lines, WAT646 was slightly different from others in the response to stripe rust (Figure 5). The leaves of WAT646 carried few spores of *Pst*. There may be two reasons for this difference. Firstly, WAT655 was a homoeologous translocation line, because the 6P segment was translocated to 6D, while the 6P segment of WAT646 was translocated to 1B [29]. Compensating translocations between homoeologous wheat and alien segments are favorable and beneficial for wheat improvement [31], so that WAT655 may display better complementary than WAT646. Secondly, the 6PS (0.81–1.00) of WAT655 was larger than the 6PS (0.86–1.00) of WAT646, so the 6P segment of WAT655 may contain more resistance genes than that of WAT646. Therefore, we localized the resistance locus on the 6P segment of WAT655. Collectively, the six translocation lines exhibit high resistance to stripe rust and can be the new stripe rust-resistant resources for resistance breeding.

*A*. *cristatum*, as a favorable genetic resource, carried various disease resistance genes [16,22,23]. The leaf rust resistance locus from *A*. *cristatum* has been transferred to common wheat [16]. Copete et al. [23] utilized the addition lines to confirm *A*. *cristatum* 2PL and 6PL carried powdery mildew resistance gene(s). Song et al. [26] mapped a leaf rust resistance locus of *A*. *cristatum* on 6PS (0.81–1.00) using *A*. *cristatum* 6P deletion lines. In this study, the stripe rust resistance locus was found to be located on 6PS (0.81–1.00). The leaf rust resistance locus and the stripe rust resistance locus from *A*. *cristatum* 6P were coincidently located on the same chromosomal segment. The translocation lines (WAT639b, WAT638a, WAT648 and WAT655) were resistant to stripe rust in this research, and they also were resistant to leaf rust. Therefore, the resistance locus mapped on 6PS (0.81–1.00) may be a broad-spectrum resistance locus, and we speculate that there may be one favorable disease resistance gene cluster on 6PS (0.81–1.00).

Molecular marker technique has been a considerably efficient and convenient method to detect the alien genomic component. A series of P genome-specific STS markers were designed through EST sequences of *A*. *cristatum* transcriptome sequences [32]. Song et al. [20] mapped 255 STS markers on the physical map of *A*. *cristatum* 6P. These markers were used to trace the 6P segments of translocation lines and deletion lines in this research. Twenty-nine STS markers were mapped on the region of the resistance locus. These 6P-specific STS markers will be useful for screening disease-resistant materials in wheat breeding, which will provide a basis for fine mapping of the wheat rust resistance locus in future work.

In recent years, many wheat varieties have lost resistance against stripe rust due to the deficiency of resistance genes and variation of *Pst* races. The emergence of two prevalent physiological races (CYR32 and CYR33) have resulted in the loss of stripe rust resistance of more varieties. However, only a few resistance genes conferred still resistance to stripe rust in common wheat, such as *Yr5, Yr10, Yr15, Yr24*/*Yr26* and *Yr50* [33,34,35,36]. Therefore, continuous exploration of new stripe rust resistance genes, particularly wide-spectrum genes, will be imperative, which will enhance the diversity of stripe rust resistance gene. Transferring stripe rust resistance genes from the wild relatives to common wheat has been an effective approach to enhance the agronomic performance of wheat [37]. Wheat-rye 1BL·1RS translocation line possesses the stripe rust resistance gene *Yr9* [7,8]. The stripe rust resistance genes from emmer wheat, such as *Yr15* [38], *Yr35*/*Lr52* [39] and *Yr30*/*Sr2* [40], have been transferred to common wheat. In this research, the stripe rust resistance locus of *A*. *cristatum* 6P has been transferred into common wheat in translocation line form. The stripe rust resistance locus is a broad-spectrum resistance locus, which can be used for genetic improvement of wheat as a new resistance source. The stripe rust-resistant lines with favorable agronomic traits can be utilized in wheat breeding as new disease-resistant wheat germplasms. The small segmental lines (WAT648, WAT655 and WAT646) will be as the basis for further exploring the stripe rust resistance locus through RNA-Sequence in future research.

In summary, the novel stripe rust resistance locus from *A*. *cristatum* 6P was located on the region 6PS (0.81–1.00) using homozygous strains and the populations. The stripe rust-resistant translocation lines will be used for wheat disease-resistant breeding as new germplasms and for fine mapping of the novel stripe rust resistance locus as the basic materials.

## 4. Materials and Methods

### 4.1. Plant Materials

The plant materials, *Triticum aestivum* cv. Fukuhokomugi (2*n* = 6*x* = 42, AABBDD), *A*. *cristatum* accession Z559 (2*n* = 4*x* = 28, PPPP, from Xinjiang, China), wheat-*A*. *cristatum* 6P disomic addition line 4844-12 (2*n* = 44), five M_5_ *A*. *cristatum* 6P homozygous deletion lines (del21, del20, del19a, del23 and del29) [26], ten BC_2_F_3_ wheat-*A*. *cristatum* 6P homozygous translocation lines (WAT639b, WAT638a, WAT639a, WAT638b, WAT657, WAT641a, WAT648, WAT655, WAT646 and WAT644) and the BC_2_F_2_ and BC_3_F_2_ populations of WAT639a and WAT639b, were utilized in this study. The detailed information of translocation lines and deletion lines was shown in Table 4. Wheat-*A*. *cristatum* 6P disomic addition line 4844-12 was acquired by distant hybridization between the *A*. *cristatum* accession “Z559” and common wheat variety “Fukuhokomugi”. All of the plant materials were preserved at the Center of Crop Germplasm Resources Research in the Institute of Crop Science, Chinese Academy of Agricultural Sciences (Beijing, China).

### 4.2. Molecular Cytogenetic Analysis

Chromosome spreads of wheat-*A. cristatum* 6P translocation lines and *A. cristatum* 6P deletion lines from root tip cells were prepared as described by Han et al. [43]. GISH was carried out as described by Cuadrado et al. [44], except that the rinsing steps were modified with 0.5× saline sodium citrate instead of 0.1× saline sodium citrate. The *A. cristatum* “Z559” P-genomic DNA and common wheat “Fukuhokomugi” genomic DNA were respectively utilized as probe and block, at a 1:40 ratio. *A. cristatum* genomic DNA was labeled by DIG-Nick Translation Mix. DIG-Nick Translation Mix and anti-digoxigenin-rhodamine (red) were purchased from Roche, Mannheim, Germany. Signals were observed using an OLYMPUS AX80 (Olympus Corporation, Tokyo, Japan) fluorescence microscope. Images were captured with a CCD camera (Diagnostic Institute, Inc., Sterling Height, MI, USA) and processed with Photoshop CS 3.0.

P genome-specific markers and 6P-specific STS markers [30,32] were used to detect translocation lines and deletion lines. P genome-specific markers can trace the P genomic component in wheat background, which were designed through specific DNA sequences distributing the entire P genome [30]. Zhang et al. [32] designed the 6P-specific STS markers according to the EST sequences from *A*. *cristatum* transcriptome. Song et al. [30] mapped 255 6P-specific STS markers on the physical map using the deletion lines and translocation lines. We used 6P-specific STS markers to trace the different 6P segments of translocation lines and deletion lines.

### 4.3. Evaluation of Stripe Rust Resistance at Adult Stage

Homozygous materials and the BC_2_F_2_ and BC_3_F_2_ populations of WAT639a and WAT639b were planted in a random complete block design with three replicates in the fields of Yangling (34°16′56.24′′ N, 108°4′27.95′′ E, Shaanxi province, China) and Xinxiang (35°18′13.71′′ N, 113°55′15.05′′ E, Henan province, China) during 2015–2016 and 2016–2017. 4844-12, Fukuhokomugi and Huixianhong were used as controls, meanwhile Huixianhong was also used as the spreader rows. Twenty grains of each line were evenly planted in 2.0 m rows, spaced 0.3 m apart. A mixture of prevalent physiological races composed of CYR32 and CYR33 was used to infect plants at wheat elongation stage.

Host responses to infection were recorded when leaves of Huixianhong were fully rusted [45]. The infection type (IT) of each plant was recorded based on 0–9 rating scale, with 0 as immune (no visible signs), 1–2 as high resistance (no or few sporulation), 3–4 as intermediate resistance (trace sporulation), 5–6 as intermediate susceptibility (intermediate sporulation), 7–8 as susceptibility (abundant sporulation) and 9 as high susceptibility (no necrosis or chlorosis; abundant sporulation). Plants with IT 0–4 were considered resistant, while plants with IT 5–9 were considered susceptible.

### 4.4. Statistical Analysis of Field Experiment

Statistical Analysis System (Version 9.2, SAS Institute, Cary, NC, USA) was used for statistical analysis in this study. Plants in each population were classified to two types according to molecular marker analysis: plants with P genome-specific markers and plants without P genome-specific markers. Chi-squared (*χ*^2^) tests for independence were used to determine the association between 6P segments and response to stripe rust.

The translocation lines were manually harvested at the maturity stage. We measured and counted the spike agronomic traits including spike length, spikelet number per spike, kernel number per spikelet, grain number per spike and thousand-grain weight. The analysis of variance was performed to test the difference between the translocation lines and the parent Fukuhokomugi in the agronomic traits.

## Figures and Tables

**Table 1:** Evaluation of stripe rust resistance of homozygous materials in two seasons.

Materials	6P Segment Size	Stripe Rust Response	No. Detected of Each Replicate	
			Xinxiang	Yangling
WAT639b	6PS arm	R	40	40
WAT638a	6PS arm	R	40	40
del21	6PS arm	R	40	30
WAT639a	6PL arm	S	40	40
WAT638b	6PL arm	S	40	40
del20	6PL arm	S	40	30
WAT657	6PS (0.00–0.15)	S	40	40
WAT644	6PS (0.00–0.20) + 6PL	S	40	40
del19a	6PS (0.00–0.15) + 6PL	S	40	30
del23	6PS (0.00–0.45) + 6PL	S	40	30
del29	6PS (0.00–0.81) + 6PL	S	40	30
WAT641a	6PS (0.53–1.00)	R	40	40
WAT648	6PS (0.59–1.00)	R	40	40
WAT655	6PS (0.81–1.00)	R	40	40
WAT646	6PS (0.86–1.00)	R	40	40

6P segment size: The comparison of the length of 6P segments and intact 6P arm. The position of the centromere was considered as 0, while the terminal end of the 6PS/6PL arm was considered as 1. The letters R and S in “Stripe rust response” column indicated plants were resistant and susceptible, respectively.

**Table 2:** Response to stripe rust in populations of 6P whole-arm translocation lines.

Materials	Type	2015–2016 (BC_2_F_2_)		Total	2016–2017 (BC_3_F_2_)		Total
		6P+	6P−		6P−	6P−	
Huixianhong			S (30)	30		S (40)	40
Fukuhokomugi			S (30)	30		S (40)	40
4844-12		R (30)	S (0)	30	R (40)	S (0)	40
WAT639b *	6PS·7AL	R (22)	S (32)	54	R (97)	S (52)	149
WAT639a	7AS·6PL	S (41)	S (29)	70	S (135)	S (54)	189

“6P+” indicated the plants contained *A*. *cristatum* 6P chromatin, while “6P−” indicated the plants did not contain 6P chromatin. The letters R and S indicated plants were resistant and susceptible, respectively. * Chi-square statistics: *p* value < 0.01.

**Table 3:** Agronomic traits for stripe rust-resistant translocation lines and their parents in 2017.

Materials	Type	Spike Length (cm)	Spikelet Number per Spike	Kernel Number per Spikelet	Grain Number per Spike	Thousand-Grain Weight (g)
4844-12		10.35 ± 0.69	23.36 ± 2.27	4.48 ± 0.51	72.96 ± 5.79	37.74 ± 0.46
Fukuhokomugi		10.24 ± 0.84	18.76 ± 1.42	4.20 ± 0.41	55.08 ± 5.85	32.98 ± 1.40
WAT638a	6PS·6AL	10.60 ± 1.16	20.79 ± 2.08 *	3.76 ± 0.57	55.71 ± 4.48	40.84 ± 3.82 **
WAT639b	6PS·7AL	10.08 ± 0.98	19.48 ± 1.58	4.22 ± 0.68	57.00 ± 3.62	35.16 ± 3.47 *
WAT641a	7A-6PS	9.50 ± 1.15	17.50 ± 1.91	4.25 ± 0.50	48.50 ± 7.93	33.08 ± 3.52
WAT648	5DS·5DL-6PS	10.05 ± 0.99	20.44 ± 1.65 *	3.77 ± 0.94	56.22 ± 5.40	46.92 ± 4.14 **
WAT655	6DS·6DL-6PS	11.26 ± 0.86 *	21.28 ± 1.50 *	4.07 ± 0.45	64.38 ± 6.65 **	38.38 ± 4.84 **
WAT646	1BL·1BS-6PS	11.52 ± 0.94 *	21.07 ± 1.59 *	4.07 ± 0.47	58.42 ± 6.15 *	44.25 ± 4.65 **

Note: * and ** denoted significant differences from Fukuhokomugi by Duncan’s multiple-range test at the probability levels of *p* = 0.05 and *p* = 0.01, respectively (analysis of variance by SAS 9.2).

**Table 4:** The detailed information of plant materials.

Materials	Zygosity	Progeny	Type	6P Segment Size
del21	Homozygous	M_5_	6PS telosomic	6PS arm
del20	Homozygous	M_5_	6PL telosomic	6PL arm
del19a	Homozygous	M_5_	6PS terminal deletion	6PS (0.00–0.15) + 6PL
del23	Homozygous	M_5_	6PS terminal deletion	6PS (0.00–0.45) + 6PL
del29	Homozygous	M_5_	6PS terminal deletion	6PS (0.00–0.81) + 6PL
WAT639b	Homozygous	BC_2_F_3_	6PS·7AL	6PS arm
	Heterozygous	BC_2_F_2_, BC_3_F_2_		
WAT638a	Homozygous	BC_2_F_3_	6PS·6AL	6PS arm
WAT639a	Homozygous	BC_2_F_3_	7AS·6PL	6PL arm
	Heterozygous	BC_2_F_2_, BC_3_F_2_		
WAT638b	Homozygous	BC_2_F_3_	6AS·6PL	6PL arm
WAT657	Homozygous	BC_2_F_3_	6AS·6PS	6PS (0.00–0.15)
WAT641a	Homozygous	BC_2_F_3_	7A-6PS	6PS (0.53–1.00)
WAT648	Homozygous	BC_2_F_3_	5DS·5DL-6PS	6PS (0.59–1.00)
WAT655	Homozygous	BC_2_F_3_	6DS·6DL-6PS	6PS (0.81–1.00)
WAT646	Homozygous	BC_2_F_3_	1BL·1BS-6PS	6PS (0.86–1.00)
WAT644	Homozygous	BC_2_F_3_	6PL·6PS-A	6PS (0.00–0.20) + 6PL

6P segment size: The comparison of the length of 6P segments and intact 6P arm. The position of the centromere was considered as 0, while the terminal end of the 6PS/6PL arm was considered as 1 (Figure 6). The arm length was measured using the software image J [41] and the fraction length value was calculated as described by Endo and Gill [42].

## Abbreviations

GISH	Genomic in situ hybridization
STS	Sequence tagged sites
EST	Expressed sequence tag
DAPI	4,6-diamino-2-phenyl indole
IT	Infection type
SAS	Statistical Analysis System
